# Characterization of the Desiccation Tolerance of *Cronobacter sakazakii* Strains

**DOI:** 10.3389/fmicb.2018.02867

**Published:** 2018-11-27

**Authors:** Xin-jun Du, Xiao-yi Wang, Xuan Dong, Ping Li, Shuo Wang

**Affiliations:** ^1^State Key Laboratory of Food Nutrition and Safety, Key Laboratory of Food Nutrition and Safety, Ministry of Education, Tianjin University of Science and Technology, Tianjin, China; ^2^Tianjin Key Laboratory of Food Science and Health, School of Medicine, Nankai University, Tianjin, China

**Keywords:** *Cronobacter sakazakii*, multilocus sequence typing, desiccation tolerance, biofilm formation, desiccation tolerance related genes

## Abstract

Strong desiccation tolerance is an outstanding feature of *Cronobacter sakazakii* and can enable the bacterium to survive in a dry food matrix (such as milk powder) for a long time. Therefore, contamination of food possessing low water activity with *C. sakazakii* can increase the risk of infection in human beings, particularly in neonates and infants. However, the mechanism underlying the desiccation tolerance property of *C. sakazakii* is largely unknown. In this study, the desiccation tolerance characteristics of 42 *C. sakazakii* strains were analyzed. Simultaneously, the sequence types and biofilm formation abilities of the strains were investigated, and their correlations with desiccation tolerance were analyzed. The results showed no significant correlation between desiccation tolerance and sequence type. However, there was a positive correlation between biofilm formation ability and desiccation tolerance. Raman spectroscopy was employed to investigate the biofilm formed by strains with distinct desiccation tolerance levels, and the results showed that the levels of polysaccharide, proteins and carotenoid might play important roles in the resistance to dry environments. In addition, 10 genes involved in osmoprotectant synthesis or transport were selected, and their differential expression in strains with diverse desiccation tolerance levels was compared to investigate whether these genes were responsible for cytoprotection in the dry environment. The results revealed a great difference in gene expression among strains with different desiccation tolerance levels, suggesting that these genes play a regulatory role in the resistance of *C. sakazakii* to dry environments. Our study provides a useful reference for follow-up studies investigating the mechanism of desiccation tolerance in *C. sakazakii.*

## Introduction

*Cronobacter* spp., classified in family *Enterobacteriaceae*, are rod-shaped, motile, non-spore-forming facultative anaerobic Gram-negative opportunistic pathogen (Farmer et al., 1980). The genus of *Cronobacter* has been classified into the following seven species: *Cronobacter sakazakii, Cronobacter malonaticus, Cronobacter condimenti, Cronobacter universalis, Cronobacter dublinensis, Cronobacter turicensis*, and *Cronobacter muytjensii* (Stephan et al., 2014). The organisms have been isolated from a wide variety of foods including cheese, meat, vegetables, grains, herbs and spices, as well as from mammals and invertebrates (Iversen and Stephen, 2003). Due to their survival in food and in the environment, these microorganisms can contact humans and cause many serious infections, such as meningitis, necrotizing enterocolitis, and septicemia. Particularly in low-birth-weight neonates, immuno-compromised neonates and infants less than 4 weeks of age, the mortality rate was about 40–80% (FAO/WHO, 2004, 2008). Additionally, cases of elderly and immunocompromised patients infected by *Cronobacter* spp. have also been reported (Iversen and Stephen, 2003). Recently, in addition to virulence factors that can be harmful to humans, the resistance of *Cronobacter* spp. to adverse environments, especially their strong desiccation tolerance and osmotic stress tolerance, has become another focus of research. These properties allow for the long-term persistence of *Cronobacter* in infant formulas and other continually unfavorable conditions (Breeuwer et al., 2003). In previous studies, members of the genus *Cronobacter* has exhibited greater desiccation tolerance. The resistance of bacterial pathogens such as *Listeria monocytogenes, Escherichia coli* O157: H7, *Salmonella enterica*, and *C. sakazakii* to dry conditions has been studied, and the results showed that *C. sakazakii* has a significantly stronger desiccation tolerance than the other organisms (Koseki et al., 2015). However, the mechanism underlying the desiccation tolerance of *C. sakazakii* is poorly understood.

In other bacteria, the mechanism underlying resistance to adverse environments is much better understood, providing a useful reference for investigating the outstanding desiccation tolerance property of *C. sakazakii*. A previous study in *E. coli* showed that when this bacterium is subjected to a hypertonic environment, a primary response of the accumulation of electrolytes, such as potassium, glutamate, etc., took place to increase the internal osmotic pressure of the cell to counteract the high external osmotic pressure, therefore preventing harm caused by the high osmotic environment (Laermann et al., 2013). The secondary response was based on the uptake or de novo synthesis of other protective compounds. In bacteria, uptaking and synthesizing compatible solutes is another important adaptive strategy to high-osmolality environments. These compounds are highly soluble molecules that are uncharged in a physiological pH environment. They are less harmful to cellular processes than electrolytes and are capable of protecting cells from adverse environments for an extended period (Feeney and Sleator, 2011). In addition, some compatible solutes, including saccharides, free amino acids and derivatives, quaternary amines and their sulfonium analogs, polyols, sulfate esters, and small peptides in *E. coli* and *Bacillus subtilis* were detected using Nuclear Magnetic Resonance (NMR) and High Performance Liquid Chromatography (HPLC) for osmoregulatory purposes (Kempf and Bremer, 1998). In further studies, an experiment performed in *E. coli* showed that intracellular trehalose could be induced by hyperosmosis and therefore promoted desiccation tolerance (Welsh and Herbert, 1999). In addition, metagenomics research on microbial responses to dry environments in diverse bacteria, such as trehalose biosynthesis, has clarified the pathways (Le et al., 2016). Furthermore, existing studies have shown that biofilms can play a protective role when bacteria are in adverse conditions. In various dry conditions, algae, fungi, archaea and bacteria mostly survive in the form of a biofilm community, in which they coexist and aggregate (Bar et al., 2002). *C. sakazakii* can form biofilms, in which cells are embedded in an excreted matrix composed of extracellular polymeric substances (EPSs) (Lebre et al., 2017), which is crucial for the protective effect of biofilms in desiccation tolerance (Bogino et al., 2013; Flemming et al., 2016; Yang et al., 2016; Fei et al., 2017). And the EPSs are primarily composed of polysaccharides, proteins, lipopolysaccharides and extracellular DNA (Bogino et al., 2013; Flemming et al., 2016). A study on *Escherichia coli* K-12 has found that EPSs involved in desiccation resistance (Danese et al., 2000). Another study on *L. monocytogenes* has found that preventing the presence of mature biofilms can reduce desiccation survival (Hingston et al., 2013). Although, there are many studies about desiccation tolerance in diverse bacteria, corresponding studies in *C. sakazakii* have not been comprehensively performed. In a previous study, compatible solutes in *C. sakazakii* were detected and analyzed by high-performance liquid chromatography (HPLC) (Breeuwer et al., 2003). However, further studies on the protective effect of trehalose and other compatible solutes in *C. sakazakii* under dry conditions have not been carried out. Previous studies indicated that colanic acid, one of the components of biofilms, and biofilm encapsulation may contribute to the persistence of *C. sakazakii* in adverse conditions (Iversen et al., 2004; Burgess et al., 2016). In addition, further investigation of the roles of biofilm in desiccation tolerance is still required to understand the mechanism underlying the prominent resistance of *C. sakazakii* to adverse conditions. Taken together, the current evidence is insufficient to explain the outstanding desiccation tolerance of *C. sakazakii*.

In order to investigate the mechanism underlying the desiccation tolerance of *C. sakazakii*, 42 strains were involved in this study and dry-condition-resistance capabilities of these strains were evaluated. Furthermore, sequence types, biofilm-forming abilities, biofilm components, and expression of 10 genes were studied, and their relationship with desiccation tolerance were analyzed and discussed.

## Materials and Methods

### Bacterial Strains and Genomic DNA Preparation

The 42 *Cronobacter sakazakii* strains used in this study. Three of them were obtained from the American Type Culture Collection (ATCC BAA-894, ATCC 29004, ATCC 12868) and other 39 were isolated from different food matrices. The isolates were mainly obtained from dairy products, whey protein products and casein products. All of the strains are listed in Table 1.

**Table 1:** The *C. sakazakii* strains used in this study.

No.	Strains	Sources	No.	Strains	Sources
1	ATCC 29004	ATCC	22	SAKA 81021	Whey powder/New Zealand
2	ATCC 12868	ATCC	23	SAKA 81104	Whole milk powder/Australia
3	ATCC BAA-894	ATCC	24	SAKA 81111	Whey protein powder/United States
4	ENS 60309-1	Skimmed milk powder/India	25	SAKA 90109	Whey protein
5	ENS 70115-2	Whey powder/France	26	SAKA 90225	Cake mix/United States
6	ENS 70208	Whey powder/India	27	SAKA 90303	Whey powder/New Zealand
7	ENS 70510	Whole milk powder/United States	28	SAKA 90309	Concentrated whey protein/Netherland
8	ENS 70817	Sample from Qinhuangdao	29	SAKA 90309-1	Concentrated whey protein/Netherland
9	ENS 70819	Whey protein powder/United States	30	SAKA 90310-1	Whole milk powder/France
10	ENS 71106	Infant formula milk powder/New Zealand	31	SAKA 90505	New Zealand
11	ENS 71123	Skimmed milk powder/Canada	32	SAKA 90807	New Zealand
12	SAKA 80220A	Peuter Groemeik/Netherland	33	SAKA 90814	Milk powder/Australia
13	SAKA 80221	Whey powder/Austria	34	SAKA 90930	Jilin Exit Inspection and Quarantine
14	SAKA 80222	Sweet whey powder/France	35	SAKA 91021	Whole milk powder/New Zealand
15	SAKA 80417-1	Casoid flour/Ireland	36	SAKA 91218	Whole milk powder/New Zealand
16	SAKA 80704	Whey powder/Netherland	37	SAKA 100531	Infant rice flour/United States
17	SAKA 80721	Skimmed milk powder/United States	38	SAKA 10319	Milk powder/China
18	SAKA 80408	Whole milk powder/Australia	39	SAKA 10119	Whole milk powder/New Zealand
19	SAKA 81013-1	Whole milk powder/Australia	40	SAKA 10208	Whole milk powder/Singapore
20	SAKA 81013-2	Whole milk powder/Australia	41	SAKA 10128-91	Bath chap/Spain
21	SAKA 81013-5	Whole milk powder/Australia	42	SAKA 110609-3	Laboratory

*Cronobacter sakazakii* strains were stored at -80°C in LB medium with 20% glycerol. A single colony from each agar plate was inoculated in 10 mL of liquid LB medium and cultured at 37°C for about 12 h until the bacterial cells in each tube reached 10^6^–10^8^ CFU/mL. Genomic DNA of all strains was extracted by a Bacterial Genome DNA Kit (Tiangen, China) according to the manufacturer’s instructions.

### Desiccation Tolerance Analysis

Desiccation tolerance analysis of the strains were performed as in previous reports with some modifications (Breeuwer et al., 2003; Barron and Forsythe, 2007; Hingston et al., 2013). After culture of the strains on plates, a single colony was cultured in 10 mL liquid LB broth until the bacterial cells reached logarithmic phase. The OD_600_ value of different bacterial culture was measured, and a 100 μL portion of the culture was transferred into each well of a 96-well microtiter plate. Subsequently, the plate was transferred into a sterile dryer with dehydrated silica gel. The dryer was placed in a sterile incubator (DHP-2042BS, Huabei, Tianjin, China), which was controlled at a constant temperature of 37°C. After 6 days of drying, the 96-well microtiter plate was removed, 100 μL/well of fresh medium was added, and the plate was cultured with 200 rpm shaking at 37°C for 3 h. The liquid in each well was transferred to a new 96-well microtiter plate, and the OD_600_ was detected. Each strain was tested in triplicate, and each test was repeated three times. The inactivation rate was calculated using the following formula to measure the desiccation tolerance of the strains:

$$\begin{array}{c} Inactivation\;rate(\%)=\frac{OD_{0}-OD_{2}}{OD_{0}}\times 100\% \end{array}$$

OD_0_ represents the initial OD_600_ of each strain, and OD_1_ represents the OD_600_ of each strain cultured for 3 h after 6-day drying treatment.

### Multilocus Sequence Typing (MLST) of *C. sakazakii*

Seven pairs of primers representing the seven housekeeping genes, including *atpD, fusA, glnS, gltB, gyrB, infB*, and *pps*, were synthesized according to the protocol on the MLST website^1^. The PCR system and conditions followed the protocol provided on the website. PCR products were sequenced by Suzhou Jinweizhi Biotechnology Co., Ltd. (Suzhou, China). To analyze the phylogenetic relationship of all 42 strains, the DNA sequence of the seven housekeeping genes were concatenated together (3,036 bp) in a certain order and used to draw the phylogenetic tree by MEGA 6.0. The relationship of the strains were analyzed by the neighbor-joining statistical method with a Tamura 3-parameter model (Baldwin et al., 2009; Joseph and Forsythe, 2012).

### Biofilm Formation Experiment

The experiment of biofilm formation is referred to in previous reports (Auger et al., 2006; Lu et al., 2012).

The overnight culture was transferred to 1 mL fresh LB medium (1:100) and cultured at 37°C until the OD_600_ value reached 0.6–0.8. 100 μL/well bacterial cells at logarithmic phase were transferred to a 96-well microtiter plate. After 3 days of static culture at 37°C, planktonic cells were removed from the liquid medium. The wells were washed three times with 150 μL of double-distilled water, and the majority of the biofilms were stained with 150 μL of 0.1% crystal violet (CV) for 30 min. Then, the unbound dye was removed, and the plates were washed with double-distilled water. Finally, the CV binding to the biofilm was dissolved in 150 μL of 95% ethanol for 30 min, and the ability to form biofilms was determined by measuring the absorbance at OD_590_ with a microplate reader. Each strain was detected three times, and fresh medium was used as a control.

### Raman Spectroscopy Analysis

Raman spectroscopy was used to examine the components of biofilm formed by strains with differing desiccation tolerance. Four strains, ENS 71106 and SAKA 110609-3 with a stronger desiccation tolerance and ENS 70819 and SAKA 90814 with a weaker desiccation tolerance, were activated and cultured until reaching exponential phase. A 50 μL portion of each strain culture was transferred to a nitrocellulose membrane (Millipore, Ireland). Next, these nitrocellulose membranes were placed on the LB agar plates and cultured at 37°C for 72 h. During this period, the LB agar plates had to be changed every 24 h (Du et al., 2012).

In this study, Raman spectroscopic analyses were performed using a Renishaw inVia Raman system (Renishaw, Gloucestershire, United Kingdom) equipped with a 785 nm near-infrared diode laser and a Leica microscope (Leica Biosystems, Wetzlar, Germany). In addition, a WITec alpha300 Raman microscope (WITec, Ulm, Germany) equipped with a UHTS-300 spectrometer was used to collect Raman spectra. Instrumental control and data collection were performed by WiRE 2.0 software (WITec, Ulm, Germany). Raman spectra were collected in the form of a simultaneous Raman shift range of 1600-600 cm^-1^ in an extended mode. For measurement in a single area, each full spectral measurement was for a 10 s integration time with 6 spectral accumulations (total integration time, 60 s). The nitrocellulose membranes on which the biofilms were formatted were placed on glass slides and then directly placed under a microscope for focus adjustment and employed for Raman spectral collection. Ten spectra were collected for each biofilm in triplicate. By creating two-dimensional images, principal component analysis (PCA) of the biofilms formed by the four *C. sakazakii* strains was performed.

### Quantitative Real-Time PCR

The two strains with a stronger desiccation tolerance (SAKA 110609-3 and ENS 71106) and the two strains with a weaker desiccation tolerance (ENS 70819 and SAKA 90814) were separately chosen for quantitative real-time PCR experiments. Ten osmotic-related genes were selected, and the expression levels of the genes were evaluated in the four strains pre- and post-dry treatment. After the *Cronobacter sakazakii* strains were cultured to exponential phase, the bacterial cells were collected by centrifugation at 6,000 rpm for 5 min at 4°C. In preparation of dry-treated samples, the bacteria were kept in a sterile incubator at a constant temperature (42°C) and a relative humidity of 43% for 1 h (Breeuwer et al., 2003). RNA was extracted using a Bacterial RNA Kit (Omega Bio-Tek, Norcross, GA, United States) and reverse transcribed into cDNA using an ImProm-II^TM^ Reverse Transcription System (Takara, Dalian, China). The cDNA was used as the template, and the 16S rDNA gene was used as an internal reference. Three biological replicates were carried out, and the fold changes in gene expression were calculated using the 2^-ΔΔCt^ method (Livak and Schmittgen, 2001). The specific primers for all ten target genes and the reference gene used are listed in Table 2.

**Table 2:** The primers specific for 10 target genes and 16S rRNA used in quantitative real-time PCR analysis.

Gene	Gene description	Amplification primer (5′→3′)
*proP* (ESA_03328)	Hypothetical protein	F: GACGCCGATTTTCCTTGAGAT
		R: GGCGACATCAGGATCACAAC
*proP* (ESA_02131)	Proline/glycine betaine transporter	F: CGCCTGTCCTGCTGTTACTCTG
		R: CGAAGCCCGCAATAGAGCC
*opuCA* (ESA_01738)	glycine/betaine ABC transporter ATP-binding protein OpuCA	F: GGACAAACAGCCGACGATTACA
		R: GCTTCTCCCGGAACGCATAT
*opuCC* (ESA_01740)	glycine/betaine ABC transporter substrate-binding protein OpuCC	F: GCGGCTGTTATGACGATGCT
		R: GACGGCTCATGCCTTTTTTC
*otsA* (ESA_01335)	Betaine aldehyde dehydrogenase	F: CGGTGAAATCGGCAATGAG
		R: CCGTTGGAGAACTGGTTGTAAT
*otsB* (ESA_01334)	Trehalose-6-phosphate-phosphatase	F: GGGCAAATGCGTAGTGGAGT
		R: GCTTCGTCGGTGAGATCGT
*kdpA* (ESA_02641)	Potassium transporting ATPase subunit A	F: CGTGGGGGCTTGGCTTTAT
		R: CGCCGCAGTTTCCAGAGTT
*kefB* (ESA_04389)	Glutathione-regulated potassium-efflux system protein KefB	F: TTACGACCAGGGCTACACACG
		R: CGGACTACGACGCACTTTATGAG
*betA* (ESA_02049)	Choline dehydrogenase	F: GCCCTACTACCGTAAGTCGGAAAC
		R: CCTGCACGCCTGCTTCTATCAT
*betB* (ESA_02048)	Betaine aldehyde dehydrogenase	F: CGCCACGGGTAACACTTT
		R: CCGCTTCCACGGCACTA
*16S rRNA*	16S rRNA	F: AGAACGCCGTCATTTACCAG
		R: GCCAAGCGATTCAACATAGTC

## Results

### Desiccation Tolerance Trial

**FIGURE 1:**
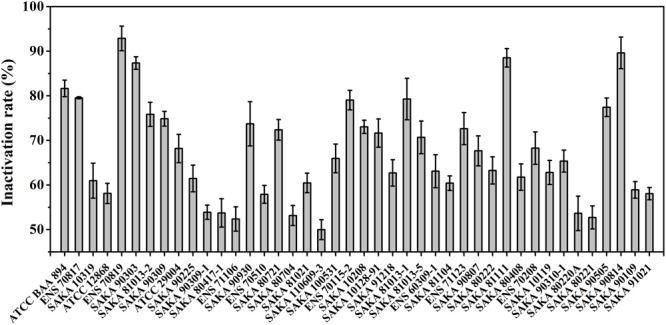
Desiccation tolerance analysis of 42 strains.

The desiccation tolerance of the strains was performed according to the formula described in the Section “Materials and Methods,” and the results are summarized in Figure 1. After 6 days of treatment in a dry environment, the inactivation rate of the seven strains was less than 55%, indicating stronger desiccation tolerant abilities. Among these strains, the SAKA 110609-3 strain showed the best capability for desiccation tolerance, and the inactivation rate was 49.98%. In contrast, the ENS 70819 strain was the weakest in terms of desiccation resistance, with an inactivation rate of 92.87%. A total of five strains showed more than an 80% inactivation rate, suggesting weak desiccation tolerance. The remaining strains (16 strains) showed moderate desiccation tolerance, with inactivation rates between 60 and 70%.

### Multilocus Sequence Typing of *C. sakazakii*

**FIGURE 2:**
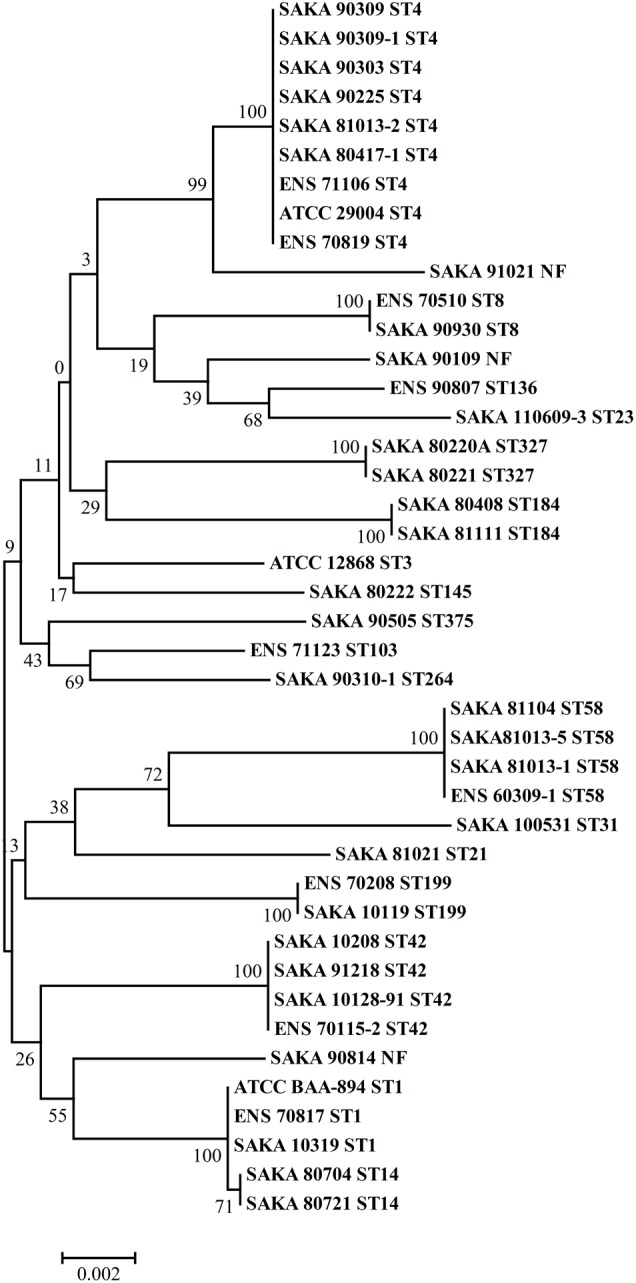
Phylogenetic tree of concatenated nucleotide sequences of the seven loci, using the neighbor-joining method with the Tamura 3-parameter model. Bootstrap values are shown for 1,000 replicates.

Multilocus sequence typing assays were performed to analyze *C. sakazakii*, and the results indicated that 20 different sequence types (STs) were identified in 42 tested strains. In addition, the STs of the strains are listed in Supplementary Table 1. Among the 20 STs, ST4 was the dominant ST (9/42), and four strains each belonged to ST42 and ST58. In addition, three strains were in the ST1 group. Two strains each were classified into ST8, ST14, ST199 and ST327. The remaining strains differed in their STs. Moreover, three strains did not match any STs in the pubMLST database of *Cronobacter* spp. Concatenated nucleotide sequences (3,036 bp) of the seven loci were used to assess all strains together in one phylogenetic tree (Figure 2). The relationship of the 42 strains were analyzed using the neighbor-joining statistical method with a Tamura 3-parameter model.

The correlation between sequence type and desiccation tolerance was analyzed. The strain with the highest desiccation tolerance (SAKA 110609-3) belonged to ST23. In addition, the strain with the weakest desiccation tolerance (ENS 70819) belonged to ST4. Analyzing the strains with an inactivation rate over 80% showed that two strains belonged to ST4 (2/9), and one strain belonged to ST1 (1/9). One of the other two strains belonged to ST184, and the remaining strain was not found in the database. Additionally, 2 ST4 strains (2/9) and 2 ST327 strains (2/2) were found to have an inactivation rate of less than 55%. The other strains were ST14, ST23, and ST8. In particular, 2 of the 9 strains of ST4, the dominant ST in our study, had weak desiccation tolerance (over 80% inactivation). In addition, another 2 of the 9 strains had high desiccation tolerance (the inactivation rate was less than 55%). The ENS 70819 strain was the ST4 strain with the highest inactivation rate, while ENS 70116, with an inactivation rate of 53.6%, was another ST4 strain with more resistance to dry environments than most strains. The great difference in the desiccation tolerance among the ST4 strains showed no significant correlation with ST type in *C. sakazakii*.

### Biofilm Formation Trial

**FIGURE 3:**
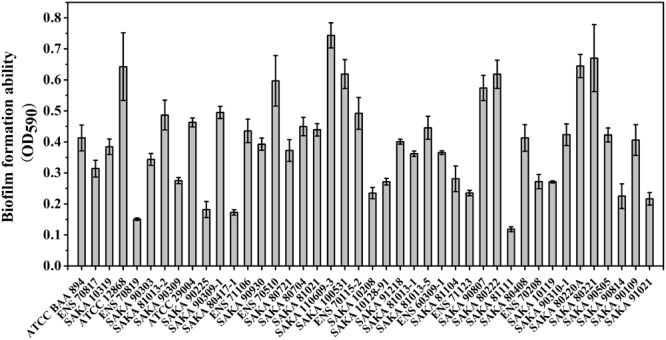
Biofilm formation ability of 42 strains.

**FIGURE 4:**
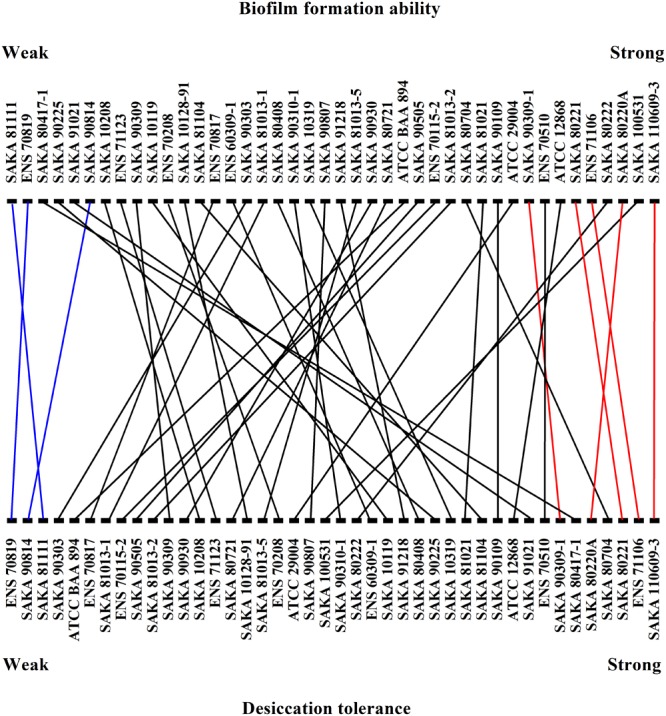
Correlation between biofilm formation ability and desiccation tolerance.

The biofilm-forming ability of the 42 strains was evaluated by CV staining. As shown in Figure 3, the biofilm formation ability of SAKA 110609-3 was the strongest, and the OD_590_ value reached 0.70. In contrast, the biofilm formation ability of the SAKA 81111 strain was the weakest, with an OD_590_ value of 0.12. Among these strains, six had higher OD_590_ values (above 0.60), suggesting that they have a higher biofilm formation ability. Four strains showed a weaker biofilm-formation ability, and the OD_590_ values were lower than 0.20. The rest of the strains exhibited a moderate biofilm formation ability. The results (Figure 4) show the correlation between biofilm formation ability and desiccation tolerance. Particularly, most of the strains with great desiccation tolerance, such as SAKA 110609-3, ENS 71106, ENS 80221, SAKA 80220A, and SAKA 90309-1 (the lines in red in Figure 4), also exhibited strong biofilm-formation abilities. In contrast, in strains with a higher inactivation rate (more than 80%), such as strains ENS 70819, SAKA 81111, and SAKA 90814 (the lines in blue in Figure 4), the biofilm formation ability was correspondingly weaker than that of most of the tested strains. Accordingly, the stains with moderate desiccation tolerance, such as SAKA 10319, SAKA 80408, SAKA 91218, SAKA 10119, SAKA 90807, and SAKA90210-1, generally showed moderate biofilm formation abilities. Taken these results together, it could be concluded that biofilm formation was positively related with desiccation tolerance.

### Comparison of Biofilm Components in Four Strains With Differing Biofilm-Formation Ability and Desiccation Tolerance

**FIGURE 5:**
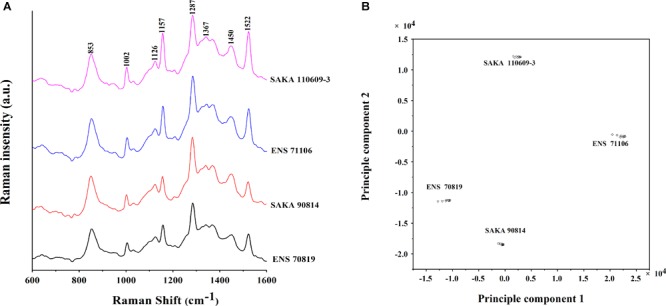
Raman spectrum analysis of biofilm formed by the four different strains. **(A)** Raman spectrum of the biofilms of the four strains (Raman Shift 1600-600 cm^-1^). **(B)** Principal component analysis (PCA) model that was based on the Raman spectrum of biofilms of the four strains.

The biofilm components of four strains with different biofilm-forming abilities and different desiccation tolerant abilities (ENS 71106, SAKA 110609-3, ENS 70819 and SAKA 90814) were studied using Raman spectroscopy. Raman peaks at different Raman shifts are shown in Figure 5A. The peaks at 1002, 1157, and 1522 cm^-1^ in two strains with a strong desiccation tolerance (ENS 71106 and SAKA 110609-3) were significantly higher than those in the other two strains with a weak desiccation tolerance (ENS 70819 and SAKA 90814). In addition, in the two strains with a strong desiccation tolerance, the peak at 1450 cm^-1^ was slightly higher than in the weak two strains. The band at 1002 cm^-1^ was characteristic of phenylalanine. In addition, the band at 1157 cm^-1^ was representative of the C-C and C-N bond stretching of proteins, while the band at 1522 cm^-1^ was associated with C-C and conjugated C = C bond stretching of carotenoids. The band at 1450 cm^-1^ was related to fatty acids (De Gelder et al., 2007; Lu et al., 2011). In addition, the peak at 1367 cm^-1^ matched the characteristics of cellulose (Lu et al., 2011) was lower in the weakest desiccation tolerance strain ENS 70819 than in the other three strains. The peak at 1367 cm^-1^ representing cellulose was the highest in ENS 110609-3 than in the four strains.

A two-dimensional PCA model was established to distinguish the four strains, and the results showed that there were differences in the main components among ENS 70819, ENS 71106, SAKA 90814 and SAKA 110609-3 (Figure 5B).

### Quantitative Real-Time PCR

**FIGURE 6:**
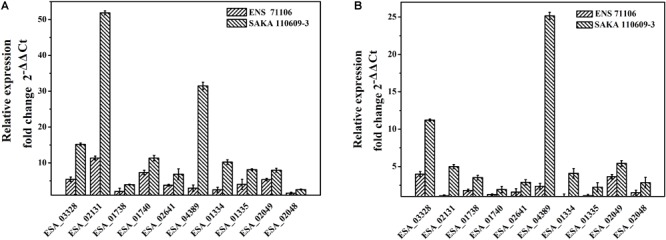
Relative expressions of 10 selected genes in strains with strong desiccation tolerance in comparison with strains with weak desiccation tolerance. **(A)** The expression levels of 10 genes in ENS 71106 and SAKA 110609-3 in comparison with those in ENS 70819. **(B)** The expression levels of 10 genes in ENS 71106 and SAKA 110609-3 in comparison with those in SAKA 90814.

According to the desiccation tolerance analysis of the 42 strains, two strains with the strongest desiccation tolerance (ENS 71106 and SAKA 110609-3) and the other two strains with the weakest desiccation tolerance (ENS 70819 and SAKA 90814) were selected for quantitative real-time PCR analysis. Ten genes associated with ions or compatible solute transport or synthesis were selected to test their expression changes between pre- and post-dry treatments. The results showed that all 10 genes in two strains with a strong desiccation tolerance were expressed at higher levels in comparison with the two strains with a weak desiccation tolerance (Supplementary Table 2). After drying treatment, proline/glycine betaine transporter (ESA_02131), glycine/betaine ABC transporter ATP-binding protein OpuCA (ESA_01738), glycine/betaine ABC transporter substrate-binding protein OpuCC (ESA_01740), trehalose-6-phosphate synthase OtsA (ESA_01335), and choline dehydrogenase BetA (ESA_02049) showed a significantly upregulated expression (*p* < 0.05). Compared with strain ENS 70819 with the weakest desiccation tolerance, all genes were upregulated in the two stains with strong desiccation tolerance (SAKA 110609-3 and ENS 71106), and the regulation in strain SAKA 110609-3 was higher than that in strain ENS 71106 (Figure 6A). Expression of the 10 genes was upregulated by 14.94-fold and 4.63-fold on average in SAKA 110609-3 and ENS 71106, respectively, in comparison with ENS 70819. Compared with the other strain with a weak desiccation tolerance (SAKA 90814), the expression levels of the 10 genes in SAKA 110609-3 were also significantly higher (average increase reaching 6.43-fold). However, in the strain with the second strongest desiccation tolerance (ENS 71106), only three genes (ESA_03328, ESA_04389, and ESA_02049) showed significantly higher expression levels in comparison with that of SAKA 90814. The remaining genes (ESA_02131, ESA_01738, ESA_01740, ESA_02641, ESA_01334, ESA_01335, and ESA_02048) showed similar expression levels in strains ENS 71106 and SAKA 90814. Based on these results, it can be concluded that the genes including ESA_03328 (encoding MFS transporter), ESA_04389 (encoding glutathione-regulated potassium-efflux system protein KefB), and ESA_02049 (encoding choline dehydrogenase BetA) are more likely to play key roles in desiccation tolerance than the other genes tested in this study (Figures 6A,B).

## Discussion

### The Relevance Between the Sequence Type (ST) and Desiccation Tolerance

Multilocus sequence typing is a method that can be used to classify various genera of *Cronobacter* spp. by analyzing the nucleotide sequences of seven housekeeping genes. The STs of 42 strains were obtained by an MLST assay. In this study, ST4 was the predominant type (9/42) in comparison with other ST types. And it is known that the most serious clinical cases of meningitis are relevant to a single ST: *C. sakazakii* ST4 (Hariri et al., 2013). In this study, ST4 was the predominant type (9/42) in comparison with other ST types. However, *C. sakazakii* ST4 showed no significantly stronger desiccation tolerance than other STs based on the results obtained in this study. Moreover, even the abilities of the nine ST4 strains to resist dry environments were obviously different (Figure 1). MLST is based on the sequence of seven genes (*atpD, fusA, glnS, gltB, gyrB, infB*, and *pps*) responsible for the basic physiological functions to classify different of *Cronobacter* isolates. However, the function of these genes was not directly related to specific characteristics such as desiccation tolerance. Therefore, it is reasonable that there was no significant correlation between the ST and desiccation tolerance of different strains.

### The Relationship Between Biofilm Formation Ability and Desiccation Tolerance

Bacterial biofilms are formed from communities embedded in a self-produced matrix of EPSs (Flemming et al., 2016). In previous studies, biofilm formation was considered an important means of protecting bacteria from long-term (even several years) survival in a dry environment (Barron and Forsythe, 2007). The correlation between desiccation tolerance and biofilm formation ability has been studied and discussed in *Salmonella* spp. (Finn et al., 2013), *L. monocytogenes* (Hansen and Vogel, 2011; Hingston et al., 2013) and *E. coli* (Chen et al., 2004; Barron and Forsythe, 2007), and the results indicate that biofilms can help bacteria avoid harm from adverse conditions. In this study, all 42 tested strains were subjected to CV staining analysis at OD_590_ to characterize the ability of biofilm formation (Figure 3). According to the results, we found that SAKA 110609-3 had the strongest desiccation tolerance, and its ability to form biofilms was much greater than that of the other strains. Furthermore, most strains with great desiccation tolerance also have a better biofilm formation ability. This result is consistent with similar studies in *L. monocytogenes* showing that biofilm formation effectively protects bacteria from adverse environmental conditions, including long-term survival in a dry environment (Hingston et al., 2013). Some researchers deemed that colanic acid and encapsulation may contribute to adherence to some surfaces but may also contribute to resistance to dry stress (Flemming et al., 2016). Studies by Scheepe-Leberlolhne and Wagner suggested that colanic acid may be an important contributor to biofilm formation and increased resistance to environmental stresses such as desiccation, heat, and pH in *Cronobacter* spp. (Scheepe-Leberlolhne and Wagner, 1986).

To further study the biofilm-forming ability among strains with different desiccation tolerance, biofilm component analysis using Raman spectroscopy was performed. Based on the Raman spectroscopy results, the main difference in the components of the four strains was appeared at peaks of 1002, 1157, and 1522 cm^-1^ for the Raman shift. The band at 1002 cm^-1^ represents phenylalanine (Manoj and Kumar, 2007), the band at 1157 cm^-1^ represents C-C and C-N bond stretching of proteins, and the band at 1522 cm^-1^ represents C-C and conjugated C = C bond stretching of carotenoids (Feng et al., 2014). In existing studies, 80% of the total biofilm composition are polysaccharide and proteins. And the macromolecules of biofilm components, such as proteins, can affect the overall properties of the biofilm (Bogino et al., 2013). In addition, carotenoids distributed on the surface of some *C. sakazakii* EPSs has been reported (Du et al., 2012). In this study, the significant difference in the biofilm components in the four tested strains were all associated with the proteins and carotenoids, showing an involvement with desiccation tolerance. In addition to polysaccharides, proteins, and carotenoids, fatty acids and cellulose are components of EPSs. In strain ENS 70819 with the weakest desiccation tolerance, the peak heights at 1450 cm^-1^ and 1367 cm^-1^ associated with fatty acid and cellulose were lower than those in the other three strains, indicating that fatty acid and cellulose were involved in producing the different biofilm-forming abilities of ENS 70819 and were therefore involved in desiccation tolerance.

### Analysis of the Relevance of the 10 Genes With Desiccation Tolerance

It is considered that when *C. sakazakii* is exposed to a low-water-activity environment, the accumulation of electrolytes increases the internal pressure and counteracts the high external osmotic pressure (Feeney and Sleator, 2011). The Kdp system and Kef system associated with electrolyte accumulation and efflux are considered the primary response regulation. In this study, *kdpA* and *kefB* were selected to demonstrate their roles in desiccation tolerance. The Kdp system is one of the transporters of K^+^, an inducible P-type ATPase with a high affinity and specificity for K^+^. *kdpA* is the potassium transporting ATPase subunit A of the *kdpABC* operon, which consists of three proteins (Heermann et al., 2014). According to the results of quantitative real-time PCR, the gene expression of *kdpA* (ESA_02641) at the mRNA level was upregulated by 1.55-fold and 2.80-fold in the two strains with strong desiccation tolerance. This result indicated that under dry conditions, K^+^ accumulation in strains with strong desiccation tolerance is a primary regulating response and is more effective than that in strains with less desiccation tolerance. However, the excess K^+^ should be effluxed to avoid the toxic effect on cells. It is reported that K^+^ efflux is required when cells undergo hypo-osmotic shock (Van der Laan et al., 2002). Glutathione-gated potassium-efflux systems (Kef) can control K^+^ efflux and prevent prolonged exposure of bacterial cells to excess K^+^ ions. In this study, the glutathione-regulated potassium-efflux system protein KefB was selected to test. As a result of quantitative real-time PCR, the expression of *kefB* (ESA_04389) was upregulated 1.19-fold and 12.67-fold in two strains with strong desiccation tolerance. In addition, this result suggested that strains with a strong desiccation tolerance have a great regulatory effect on protecting strains in high K^+^ concentrations.

Potassium and glutamate serve as temporary osmoprotectants, but a high ion concentration in cells for an extensive period can bring great harm to cells. *C. sakazakii* can accumulate compatible solutes by uptake from the environment or by self-synthesis to adapt to hypertonic conditions (Feeney and Sleator, 2011). Compatible solutes, such as trehalose and glycine betaine, participate in the adaptation strategy in most xerotolerant microorganisms (Santos and Da Costa, 2002). In this study, the expression of two genes in the ProP system (ESA_02131 and ESA_03328) and two other genes (ESA_01738 and ESA_01740) encoding OpuCA and OpuCC were studied. The ProP system, OpuCA and OpuCC are the major osmoprotectant absorption system in *C. sakazakii* (Sleator et al., 2001; Feeney et al., 2014). The ProP system involves the transport of proline, glycine betaine, and ectoine, and proteins OpuCA and OpuCC are involved in the transport of choline and carnitine. It was shown that the expression of *proP* (ESA_02131) in *C. sakazakii* BAA-894 was upregulated under osmotically stressful conditions (Feeney et al., 2014). In the present study, the four genes in the strains with strong desiccation tolerance were upregulated after 1 h of exposure to drying conditions. However, the situations were different in weaker desiccation tolerance strains. In addition to *opuCA* (ESA_01738), the tested genes in strain ENS 70819 (weak desiccation tolerance) showed a downregulated expression in comparison with the other 3 strains. Taken together, strains with significantly differing desiccation tolerance usually exhibited a different regulatory ability of uptaking and transporting osmoprotection between strains with different desiccation tolerance abilities.

In addition to being transported into cells, a compatible solute such as endogenous trehalose can be synthesized in *C. sakazakii* cells (Hu et al., 2017). The trehalose biosynthesis pathway in *C. sakazakii* is similar to the trehalose-6-phosphate synthase/phosphatase (OtsA-OtsB) pathway in *E. coli*. Glucose is converted to glucose-6-phosphate. Then, glucose-6-phosphate is converted to trehalose-6-phosphate, which is subsequently converted to trehalose. The gene *otsA* encoding the trehalose-6-phosphate synthetase is strongly stimulated by the accumulation of K^+^, glutamate and salts of other monovalent cations in the primary response (Strom and Kaasen, 1993). In this study, quantitative real-time PCR results showed that *otsA* and *otsB* (ESA_01334) were upregulated in mRNA levels in the three strains except for strain ENS 70819. Breeuwer et al. (2003) found the accumulation of trehalose in dried stationary cells of *C. sakazakii*, and no accumulation was detected in dried exponential cells (Breeuwer et al., 2003). In this study, the cells were cultured to exponential phase, and the regulatory response of the genes relevant to trehalose synthesis was observed in *C. sakazakii* using quantitative real-time PCR. Furthermore, the upregulation level of *otsB* was higher than that of *otsA*, most likely because during the synthetic process by which trehalose-6-phosphate is converted to trehalose, more trehalose-6-phosphate-phosphatase was required.

Choline is an important compatible solute involved in resisting harm from adverse environments. Proteins BetA and BetB are associated with converting choline to betaine in *E. coli.* After being transported into cells, choline is converted to betaine aldehyde by choline dehydrogenase (BetA). In addition, betaine aldehyde is transformed to betaine, catalyzed by betaine aldehyde dehydrogenase (BetB) (Landfald and Strøm, 1986). BetA and BetB have been identified in *C. sakazakii* and the function verified in response to desiccation by comparative proteomic analysis (Hu et al., 2017). In this study, except for strain ENS 70819, the two genes *betA* (ESA_02049) and *betB* (ESA_02048) were all upregulated in the other three strains. Furthermore, the upregulation in the strains with a strong desiccation tolerance was more significant than that in the weaker ones, showing that the betaine synthesis has already started after 1 h of drying treatment and that the synthesis in strains with a strong desiccation tolerance was more active.

In summary, the regulatory mechanisms of desiccation resistance, such as K^+^ accumulation and efflux as well as the uptake and synthesis of compatible solutes, begin after 1 h of drying. Moreover, the expression of related genes at the mRNA level showed differences among *C. sakazakii* strains with different desiccation tolerance levels. The levels of these genes in strains with strong desiccation tolerance were higher than those in the strains weak desiccation tolerance. The higher levels of these genes may make strains more resistant to dry environments.

## Conclusion

In this study, 42 *C. sakazakii* strains were evaluated for desiccation tolerance and subgrouped by MLST analysis. No significant correlation was observed between the ST and the desiccation tolerance of the strains. By contrast, biofilm formation ability and desiccation tolerance showed a positive correlation. Quantitative real-time PCR analysis indicated that 10 genes played major roles in the primary and secondary responses in desiccation tolerance. This study provides a useful reference for further study of the mechanism underlying the strong resistance of *C. sakazakii* to dry conditions, at the strain selection level and the gene selection level.

## Data Availability Statement

All datasets [GENERATED/ANALYZED] for this study are included in the manuscript and the Supplementary Files.

## Author Contributions

X-JD and X-YW contributed to the conception and the design of the study, performed the experiments, and the writing and editing of the manuscript. PL, X-JD, and SW contributed to conceived and designed of the work. XD made a great contribution by performing the experiments, by being involved in the process of the experimental design and the writing of the manuscript. All authors agreed to be accountable for the content of the work contributed to the conception of the study.

## Conflict of Interest Statement

The authors declare that the research was conducted in the absence of any commercial or financial relationships that could be construed as a potential conflict of interest.

## Footnotes

1 https://pubmlst.org/cronobacter/
